# Incentivizing community health workers for scaling up mental health care in rural communities in India: A critical look at principles that work

**DOI:** 10.3389/frhs.2023.1119213

**Published:** 2023-02-21

**Authors:** Mercian Daniel, Pallab K. Maulik

**Affiliations:** ^1^ Research, The George Institute for Global Health, New Delhi, India; ^2^ Faculty of medicine, University of New South Wales, Sydney NSW, Australia; ^3^ Prasanna School of Public Health, Manipal Academy of Higher Education, Manipal, Karnataka, India; ^4^ Department of Brain Sciences, Imperial College London, London, United Kingdom; ^5^ Research, The George Institute for Global Health, London, United Kingdom

**Keywords:** community health workers (CHW), incentive, mental health programs, mental health services, India, developing countries

## Abstract

Given the low availability of trained mental health professionals, there is evidence on task sharing where basic mental healthcare can be provided by trained community health workers (CHWs). A potential way to reduce the mental health care gap in rural and urban areas in India is to utilize the services of community health workers known as Accredited Social Health Activists (ASHAs). There is a paucity of literature that have evaluated incentivizing non-physician health workers (NPHWs) vis-à-vis maintaining a competent and motivated health workforce especially in the Asia and Pacific regions. The principles around what works and does not work in terms of a mix of incentive packages for CHWs, while providing for mental healthcare in rural areas have not been adequately evaluated. Moreover, performance-based incentives which are receiving increasing attention from health systems worldwide, though evidence on the effectiveness of these incentives in Pacific and Asian countries is limited. CHW programs that have shown to be effective rely on an interlinked incentive framework at the individual, community, and health system levels. Drawing learnings from the past eight years in implementing the SMART (systematic medical appraisal, referral and treatment) Mental Health Program in rural India, we critically examine some of the emerging principles in incentivizing ASHAs while scaling up mental healthcare in communities using a systems approach.

## The context of community health workers or non-physician health workers

The community health workers' (CHWs) or the ASHA (Accredited Social Health Activists) program was formalized and introduced in India by the Ministry of Health and Family Welfare as part of the National Rural Health Mission in 2005 (1). About one million ASHAs, the largest group of CHWs in the world, act as a bridge between the health staff and rural community members and form the backbone of India's primary healthcare system. The Indian government contracts ASHAs in each village with one ASHA servicing a population of about 1,000 individuals. ASHAs are recruited by the Panchayat (local village-level government) and are the brides of the village who are generally educated upto lower secondary level (8–10 standard). They receive basic government training in providing maternal and childcare and were historically involved in providing only such care, but now are increasingly involved in screening and caring for other health conditions too given the rise in burden of non-communicable disorders. They are contractual workers remunerated based on their performance and often held accountable as if they were fulltime permanent employees (1, 2) and the government allows them to work on other projects during their free time. Many non-government organizations use this opportunity to involve ASHAs on different projects. Generally non-government organizations reimburse them for time spent by them on their projects, however such is often not the case for many government-related activities where they are paid a consolidated fee with the expectation that they will work on other government projects, too. This has led to a greater discussion within the community and ASHAs about their remuneration structure and workloads, and a more recent push by ASHAs to consider them as government staff with additional benefits and job securities. A distinction between CHWs and other professional staff is outlined in Supplementary Table S1.

## Incentives for NPHWs: what works and does not

The different forms of incentives to influence work related behavior of health workers in low-and middle-income countries (LMICs), have primarily been financial in nature partly because of their low-income levels compared to developed industrial countries (3). Policy makers believe that monetary incentives may motivate CHWs to join the health workforce, retain and sustain their performance. The typologies of CHWs incentives are mostly extrinsic and are outlined in Supplementary Table S2.

### Financial incentives

There are some CHW programs that have successfully used monetary incentives effectively (4, 5). However, using financial incentives to improve performance and retention of health workers are often accompanied by several issues (6). A common issue that ASHAs face are that they are often not paid regularly and on time and there may be considerable delays (7). Researchers have suggested that alternative payment terms, such as “field allowances”, “transport allowances”, “travel allowances”, “per diem” that are tied to discreet tasks which are simple to measure and track could be used, too (7). Financial incentives can also increase preexisting inequity among different cadres of healthcare workers and among the CHWs' themselves. However, if there is no uniform policy that governs and regulates ASHAs allowances across India, it leads to situations where the ASHAs are dissatisfied with what they get and demand revisions in their remunerations (2). Similar experiences have been reported from other countries (8, 9).

Another form of monetary incentives is paying CHWs in-kind rather than in-cash. In-kind incentives can be in the form of material items that many organizations provide, such as bags to carry supplies, agriculture tools, raincoats, backpacks, supplies for home improvement, educational materials, herbal plants, and fruit trees (7). But material incentives that are given in too many forms and too frequently may not always serve the purpose of sustaining performance levels in the long term (7). CHWs can also be incentivized by giving preferential benefits, like access to credit programs, literacy classes, or first-in-line treatment at health posts (10, 7).

### Non-financial incentives

Financial or in-kind incentives that are given to CHWs in itself are not enough to sustain and hold CHWs' interests and motivation. Therefore, other types of incentives, often non-material are critical to CHWs' job satisfaction. These intangible incentives include a belongingness and a working relationship with the health department and its staff, a sense of identity, scope and opportunities for individual growth, training, and peer support. Perhaps one of the most important non-monetary incentives is a respectable relationship with the community. CHWs need to feel supported and appreciated by the health system (11). Regular visits by different donors and NGOs where CHWs have limited contact with the health system may help in sustaining their commitment to deliver healthcare services (12).

Supervision that is provided with an honest intention to mentor and build capacity is appreciated by CHWs. Alternatively, what does not work is weak, inadequate, and inconsistent supervision and is related to low rates of retention among CHWs (13–16). Acquiring and learning new skills is one of the primary reasons CHWs volunteer. It helps them to advance their career and receive higher monetary allowances (4). The status of CHWs in the community is enhanced if they have the right skills that is valued by community members (16). The methods that trainers use to impart knowledge and skills to CHWs are critical for these to be translated into real life and act as a major factor that incentivizes their sustained participation. Continuous training in the form of refresher trainings has been cited by Frankel (13) as an essential requirement for an effective CHW program. A comprehensive CHWs training model includes a combination of training and supervision strategies with active engagement of an NGO, health department and community trainers and supervisors. Both formal and informal group meetings are seen as strategies to motivate and incentivize CHWs through peer support mechanisms (17). Projects in Colombia, Mozambique, Nepal, and Uganda have shown peer support is as important to CHW performance as supervisory feedback (18–20). There are numerous instances of CHWs' association in many countries. These associations serve the purpose of raising funds for themselves, organize training programs and advocate for health as well as CHWs' rights to the government (7). Providing an identity with clear roles and responsibilities and mechanisms to support this is a strong non-monetary incentive for CHWs. Job aids that help a CHW to perform required tasks, strengthen skills, and which provide autonomy and authority, boost confidence, and increase competence to fulfill their roles (7), have been found to be beneficial. Opportunities for personal growth and development has been flagged as a major incentive for CHWs.

### Combining a mix of and innovative incentives for community health workers

CHW programs that have shown to be effective rely on an interlinked incentive framework at the individual, community, and health system levels (7, 21). The incentives must be based on the local context and the organization's structure; culture and institutional capacity; wider social values and expectations; ease of implementation and monitoring; cost and timeframe for the package to take effect; and the sustainability of the package (21). Performance based non-financial incentives such as career development, training opportunities and fellowships and performance-based financial incentives, which were found to be effective among some cadres of managerial healthcare workers could also work for CHWs (22, 23). However, it is critical that the introduction of performance-based incentive programs for health workers may not be successful if there is a dearth of resources to finance the scheme and monitor its implementation (24). As CHWs typically work in teams, many programs have experimented with group incentives. However, evidence on this tend to be inconclusive coming primarily from high-income countries as a result of competing interests between group and individual needs (25). In Pacific and Asian countries, the incentive structure for CHWs must be simple enough to be easily understood by them and managed as well as monitored by support staff (21, 23, 26).

## Principles that work from our experience

The principles around what works and does not work in terms of monetary and non-monetary incentives is true across all disciplines of health care delivery. Below we use examples of providing mental health care using ASHAs in rural settings as a case study. Since 2014, George Institute for Global Health India has implemented a mental health services delivery program called the Systematic Medical Appraisal, Referral and Treatment (SMART) Mental Health Program in India. Initially a large pilot project was conducted across 42 villages covering about 50,000 inhabitants in rural Andhra Pradesh, in South India. The program involved technology-enabled mental health service delivery for depression, anxiety and increased suicide risk; an anti-stigma campaign to raise awareness about those mental health conditions and reduce stigma related to mental health service use; and train primary health workers to identify and manage individuals suffering from such conditions (27, 28) That project has been scaled up as a cluster randomized trial to rural adult communities in two states of north (Haryana) and south India (Andhra Pradesh). It was implemented across 44 primary health centres that catered to 133 villages covering more than 200,000 adults (29). It is now also being implemented across 60 urban slum clusters in the cities of New Delhi (Delhi) and Vijayawada (Andhra Pradesh) as part of another cluster randomized trial (30). Drawing on learnings from the past eight years in implementing the SMART Mental Health Program in rural India and engaging with over 500 ASHAs, the subsequent section critically examines some of the emerging principles in incentivizing ASHAs while scaling up mental healthcare in rural communities.

### Monetary factors that motivate individual CHWs

The decision to establish the amount of financial incentives for CHWs were initially based on the incentive structure of the National Tuberculosis Program (31). The principle that guided us to adopt the existing government incentive structure to our program was based on the close resemblance of the tasks and responsibilities that the ASHAs would undertake to those that they undertook as part of the government-run National Tuberculosis Program. CHWs were familiar with the existing incentive structure that included incremental amounts based on their performances tied to discreet tasks, such as initial visit and referral to primary care doctors, subsequent follow-up and monitoring of treatment adherence. These were simple to measure, track, and monitor. More importantly, this financial incentive structure was readily understood by the ASHAs, and they perceived that the amounts being paid were commensurate to the tasks expected out of them. However, there were negotiations with the ASHAs on determining the right amount of incentives and their feedback was taken deciding what the incentive amounts would be. We could sustain the motivation of ASHAs to engage in the SMART Mental Health Program by ensuring that incentives were paid regularly directly into their accounts and on time while keeping the process transparent. Paying these incentives on time before any local festival increased the motivation levels of ASHAs as well as the credibility of our institute. ASHAs were monetarily incentivized to attend training programs by reimbursing their travel costs and paying a daily allowance to cover for time. Another in-kind monetary incentive that we provided to ASHAs were in the form of a handbag where they could keep the digital tablet device and a handy flipbook used as job aids for the intervention. During COVID, we provided them with supplies of sanitizers, masks and protection kits which supplemented those provided by the government and covered for any gaps in supplies from the government.

### Non-monetary factors that motivate individual community health workers

CHWs (ASHAs) were supported to perform confidently on a new topic and disease condition by providing them with trainings that intensively covered the subject matter using interactive multimedia methods to improve retention of content. Booster trainings were also conducted mid-way during the intervention to refresh their skills and support their work. Peer support in the form of directly connecting better performing ASHAs with those who needed improvement was done on a regular basis through WhatsApp groups and to help them learn from each other's experiences. As part of our program fidelity measures, we regularly assessed competency levels of ASHAs by administering pre and post competency training questionnaires. This helped us to appraise CHWs on their acquired competency skills and assist those who needed additional support. Since all activities by ASHAs were conducted using electronic tablets, project staff scrutinized their performances on the server-side backend data on regular basis and provided feedback to them routinely either by phone or through in-person meetings. This was also seen by ASHAs as a positive confidence building measure that helped them to work more efficiently, which in turn was linked to their overall performance and activity-based remuneration. Acquiring knowledge and skills about managing mental disorders was appreciated by ASHAs and the community *per se*. ASHAs also felt using tablets helped them to reduce time spent writing out notes in multiple forms and empowered them (32, 33).

A systems approach outlined in Table 1 could be used to understand CHWs incentives. Other non-monetary incentives that may motivate CHWs engaged in mental health programs in rural regions of India may be that they perceive themselves to be altruistically contributing to the greater good of their own community by helping members overcome mental health problems and especially prevent suicide. This enhances the communities' appreciation of CHWs’ efforts as they are able to see these bringing about visible changes and benefitting community members as we saw in the SMART Mental Health Program (32, 33). This acts as a non-monetary incentive for CHWs as they are recognized and respected by the community for their work. Using digital tablets to screen and manage community members with common mental disorders may also act as enhancing their perceived status in the community and hence empowering them in this process (33).

**Table 1:** A systems approach to CHWs’ incentives and disincentives.

Motivating Factors	Incentives	Disincentives
Monetary factors that motivate individual CHWs	• Satisfactory remuneration/Material Incentives/Financial Incentives • Commensurate with workload • Incremental increase in material benefits • Possibility of future paid employment	• Inconsistent remuneration • Non-commensurate with workload • Change in tangible incentives • Inequitable distribution of incentives among different types of community workers
Nonmonetary factors that motivate individual CHWs	• Altruistic contribution to community • Acquisition of valued skills through trainings • Personal growth and development • Accomplishment and empowerment • Peer support and excellence • CHW associations • Identification (badge, shirt) and job aids • Status within community • Preferential treatment • Flexible and minimal hours with clear role • Active handholding and supervision	• Person not from community • Peer competition or incompetence • Inadequate refresher training • Inadequate supervision • Excessive demands/time constraints • Lack of respect from health facility staff
Community-level factors that motivate individual CHWs	• Community recognition and respect of CHW work • Community involvement in CHW selection • Community organizations that support CHW work • Community involvement in CHW training • Community information systems	• Inappropriate selection of CHWs • Lack of community involvement in CHW selection, training, and support • Negative behavior and attitude from community members
Factors that motivate communities to support and sustain CHWs	• Witnessing visible changes • Contribution to community empowerment • CHW associations • Successful referrals to health facilities	• Unclear role and expectations (preventive vs. curative care) • Inappropriate CHW behavior • Needs of the community not considered
Factors that motivate Government health staff to support and sustain CHWs	• Policies/legislation that support CHWs • Witnessing visible changes • Funding for supervisory activities from government and/or community	• Inadequate staff and supplies • Non-performance of CHW

Source: Adapted from Bhattacharyya et al. 2001 (7).

## Conclusion

While there are several approaches to improve the motivation and retention of CHWs by incentives, there is a lack of conclusive evidence on what works best. There is a need to examine and analyze this further in order to better understand factors that are associated with CHWs motivation and retention in relation to the different types of incentives or a combination of packages of incentives. A CHWs program should combine incentives targeted at different parts of the systems, financial or non-financial factors that affect the individual CHW, community factors that encourage and support CHWs, and health system factors that support CHWs. Policy and program planners can draw on the public health community's extensive experience with CHW programs. This short reflective paper, we hope will help public health and implementations science researchers and program implementers apply the principles of incentivizing CHWs that appear to work on the ground. These principles would in the long run have implications for the retention and performance of such an important cadre of health workers, which will result in the improvement of the health status of communities.

## Data Availability

The original contributions presented in the study are included in the article/**Supplementary Material**, further inquiries can be directed to the corresponding author/s.
