# Supplementary material for: Incentivizing community health workers for scaling up mental health care in rural communities in India: A critical look at principles that work
Source: Front Health Serv. 2023 Feb 21;3:1119213. doi: 10.3389/frhs.2023.1119213 (PMC10012787; doi:10.3389/frhs.2023.1119213)
Supplement: Supplementary file 1 [file Table1.docx]

**Supplementary Table 1. Comparison of CHWs with professional health staff**

| ***Auxiliary nurses or health technicians***  ***(professional health staff)*** | ***Health promoters or village health***  ***workers (volunteers from the community)*** |
| --- | --- |
| - Primary education plus 1–2 years of training - From outside the community - Employed full time - Salary usually paid by the program   (not by the community) | - Third grade education plus 1–6 months of training - From the community - Women or referred to bride of the community - Employed part time on contractual basis - Remuneration supported by community institutions - Supported by farm labor or other community help - May be traditional healers |

*Source:* Adapted from Walt, G. Community Health Workers: Policy and Practice in National Programs. EPC Publication No. 16. London: The Evaluation and Planning Centre, London School of Hygiene & Tropical Medicine, 1988.

**Supplementary Table 2. Typology of incentives for healthcare workers**

| **Individual incentives** | **Organizational incentives** | **Environmental incentives** |
| --- | --- | --- |
| *Financial*  Salary  Pensions  Illness, health, accident,  and life insurance  Travel and transport  allowances  Childcare allowance  Rural location allowance  Heat allowance  Retention and professional allowances  Subsidized meals, clothing, and  accommodation  *Nonfinancial*  Vacation days  Flexible working hours  Access to training and  education  Sabbatical and study leave  Planned career breaks  Occupational health  Functional and  professional autonomy  Technical support and  feedback systems  Transparent reward  systems  Valued by the organization | *Internal*  Autonomy  Accountability  Market exposure  Financial responsibility  *External*  Governance  Public finance policy  Regulatory mechanisms | Amenities  Transportation  Job for spouse  School for children |

*Source:* Adapted from Zurn, P. 2003. “Incentives for Human Resource Management.” Paper presented at the Workshop on Human Resource for Health Development: The Joint Learning Initiative, Veyrier-du-Lac, France, May 8–10.
